# Alleviating central venous oxygen saturation (S_cv_O_2_): a new approach of kidney protection after cardiac surgery?

**DOI:** 10.1186/s13054-015-1079-2

**Published:** 2015-10-21

**Authors:** Patrick M. Honore, Rita Jacobs, Inne Hendrickx, Elisabeth De Waele, Duc Nam Nguyen, Herbert D. Spapen

**Affiliations:** ICU Department, Universitair Ziekenhuis Brussel—Vrije Universiteit Brussel, 101 Laarbeeklaan, 1090 Jette, Brussels Belgium

In a recent article, Balzer et al. [1] reported an unexpected relationship between an initial central venous oxygen saturation (S_cv_O_2_) >80 % on ICU admission and increased morbidity and mortality in cardiac surgery patients. Interestingly, as compared with low (<60 %) or normal (60–80 %) S_cv_O_2_ levels, values above 80 % were associated with more acute kidney injury (AKI) and a higher hemodialysis need. This goes against common belief that a higher S_cv_O_2_ is a prerequisite for more optimal organ, and in particular kidney, protection.

Transient brief episodes of upper arm ischemia after induction of anesthesia reduce the rate of AKI and the need for renal replacement therapy among high-risk cardiac surgery patients. This so-called “remote ischemic preconditioning” is thought to engage adaptive natural defense mechanisms that protect the kidneys against the subsequent significant inflammatory and ischemia/reperfusion stress caused by cardiac surgery [2]. Theoretically, keeping S_cv_O_2_ levels within a “low–normal” range in the early post-cardiac surgery period could act as a surrogate for ischemic preconditioning. In contrast, “boosting” S_cv_O_2_ by excessive transfusion, inotropic support or oxygenation might abrogate this protective mechanism [3]. The provocative findings of Balzer et al. highly encourage further research in this area.

## Abbreviations

AKI: Acute kidney injury
S_cv_O_2_: Central venous oxygen saturation
