# Supplementary material for: Alleviating central venous oxygen saturation (ScvO2): a new approach of kidney protection after cardiac surgery?
Source: Crit Care. 2015 Oct 21;19:359. doi: 10.1186/s13054-015-1079-2 (PMC4618129; doi:10.1186/s13054-015-1079-2)
Supplement: Related texts

# Reply: Authors’ response

**Authors:** Felix Balzer, Sascha Treskatsch, Michael Sander

Professor Honore and colleagues added a very interesting aspect to the discussion of initial ScvO_2_ assessment after a patient’s postoperative admission to the ICU. As published by Zarbock et al. [2], remote ischemic preconditioning is associated with reduced incidence of AKI and use of renal replacement therapy. This remarkable finding requires further investigation. The design of our approach, however, does not allow us to draw conclusions related to this aspect. In our study, patients with a presumably “safe” initial ScvO_2_ above 80 % showed a higher incidence of renal dysfunction including an increased rate of renal replacement therapy. Also, these patients had the highest in-hospital mortality and the highest mortality during 3-year follow-up. Since pre-existing chronic hemodialysis with arterial–venous shunts is known to lead to increased venous saturation, we excluded such patients from a subanalysis in order to account for this bias, but results remained substantially unchanged.

In the group of patients with high ScvO_2_, we found a significantly higher occurrence of epinephrine application. Consequently, it cannot be ruled out that the use of epinephrine aggravates the shunting effects in postoperative cardiac surgery patients. Nonetheless, the lower oxygen extraction in the high ScvO_2_ group suggests that tissue hypoperfusion may be another important component explaining the significantly increased rate of organ dysfunction and the higher mortality.

At our hospital, we have developed an algorithm to guide treatment by advanced hemodynamic monitoring in patients that present with an oxygen extraction dysfunction as reflected by a high ScvO_2_ in the context of a postoperative “sepsis-like” syndrome. Currently we are evaluating the effect of these measures on patient outcome.
